# Supplementary material for: Frequent Lucid Dreaming Is Associated with Meditation Practice Styles, Meta-Awareness, and Trait Mindfulness
Source: Brain Sci. 2024 May 14;14(5):496. doi: 10.3390/brainsci14050496 (PMC11120098; doi:10.3390/brainsci14050496)
Supplement: Supplementary file 1 [file brainsci-14-00496-s001.zip › brainsci-2989987-supplementary.pdf]

Supplementary Material

Table S1

Descriptive statistics of RMMtm items and factor loading matrix using principal components analysis (Oblimin rotation).

RMMtm Item	Mean (SD)	Factor 1: Mindful Relaxation and Focus	Factor 2: Mindful Transcendence
RMM 1: Relaxed Muscles	4.59 (1.83)	.792	
RMM 2: Relaxed Breath	5.10 (1.74)	.793	
RMM 3: Ease and Peace	4.80 (1.69)	.928	
RMM 4: Feeling Refreshed	4.53 (1.63)	.879	
RMM 5: Pleasant Mind Wandering	4.71 (1.87)	.727	
RMM 6: Centered, Grounded	4.59 (1.74)	.839	
RMM 7: Quiet and Present	4.21 (1.81)	.742	
RMM 8: Accepting	4.48 (1.82)	.798	
RMM 9: Letting Go	4.53 (1.83)	.739	
RMM 10: Effortless	4.15 (1.83)	.780	
RMM 11: Present Moment	4.69 (1.68)	.848	
RMM 12: Calm Observer	4.24 (1.89)	.670	
RMM 13: Focused, Clear, Aware	4.22 (1.77)	.641	
RMM 14: Curios, Fascinated	5.01 (1.74)	.580	
RMM 15: Vivid Experience	3.87 (1.88)	.454	.396
RMM 16: Purposeful	4.22 (1.95)	.538	
RMM 17: Motivated	4.34 (1.74)	.791	
RMM 18: Ready for the Day	4.46 (1.72)	.795	
RMM 19: Creative and Inspired	4.55 (1.62)	.684	
RMM 20: Focus on One Thing	4.60 /1.78)	.817	
RMM 21: Optimistic	4.91 (1.70)	.800	

RMM 22: Compassion	4.77 (1.73)	.599	
RMM 23: Thankful	5.01 (1.75)	.548	
RMM 24: Awe and Wonder	4.46 (1.77)	.484	.359
RMM 25: Prayerful	3.11 (2.05)		.749
RMM 26: Something Greater	3.46 (2.10)		.832
RMM 27: Concerns seemed Small	3.92 (1.91)		.602
RMM 28: Timeless, Boundless, Infinite	3.45 (2.02)		.789
RMM 29: Connected with Everything	3.26 (1.93)		.784
RMM 30: Deeper Truth	3.17 (2.02)		.853
RMM 31: Experience Beyond Words	2.47 (1.78)		.853
RMM 32: Profound Mystery	2.85 (1.93)		.873
Eigenvalue		12.856	5.848
Percentage of Explained Variance		0.402	0.183
Cumulative Percentage of Explained Variance		0.402	0.584

Note: $n = 291$.

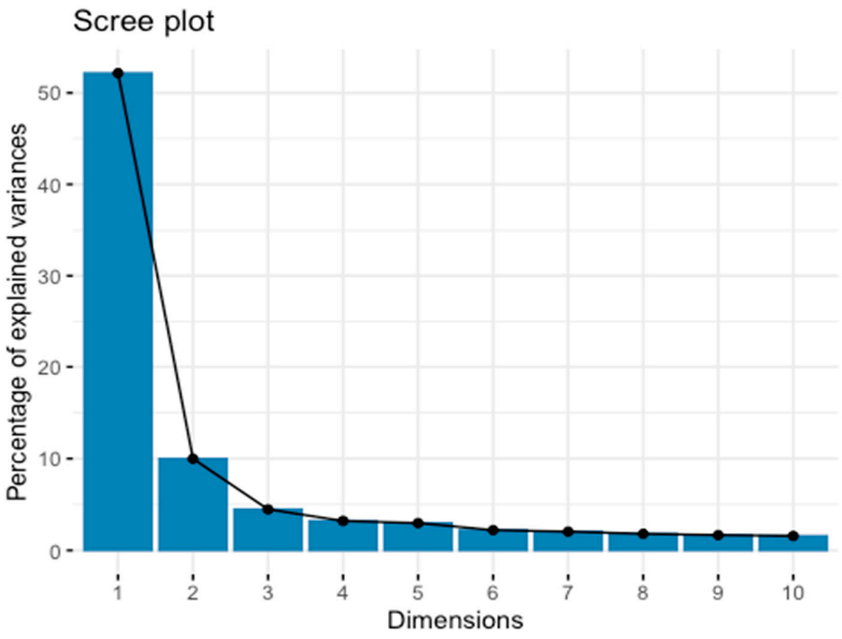

Figure S1 Scree plot of PCA result, indicating two factors.

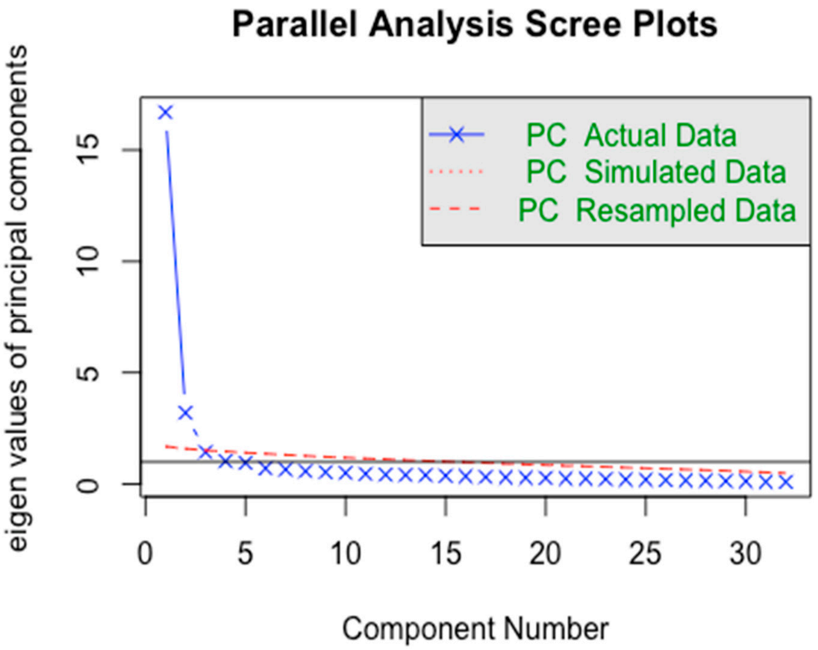

Figure S1 Parallel analysis plot of PCA, indicating two factors.
